# High performance eco-friendly free abrasive machining using an additively fabricated tool and PCD based slurry

**DOI:** 10.1038/s41598-025-16363-0

**Published:** 2025-08-22

**Authors:** Dawid Zieliński, Mariusz Deja, Wit Grzesik, Krzysztof Żak

**Affiliations:** 1 Department of Manufacturing and Production Engineering, Faculty of Mechanical Engineering and Ship Technology, Gdańsk University of Technology, Gabriela Narutowicza Street 11/12, Gdansk, 80 - 233 Poland; 2 Opole University of Technology (former), ul. Prószkowska 76, Opole, 45 - 758 Poland; 3 Department of Manufacturing Engineering and Production Automation, Faculty of Mechanical Engineering, Opole University of Technology, ul. Mikołajczyka 5, Opole, 45 - 271 Poland

**Keywords:** Lapping, Selective laser sintering (SLS), Diamond grains, Abrasive machining, Engineering, Mechanical engineering

## Abstract

This paper presents the results from lapping experiments conducted with the use of a lapping plate fabricated by the selective laser sintering (SLS) from the polyamide PA2200 powder. The minimum quantity of diamond grains (grade D107) and paste (grade SD 28/20) were uniformly distributed on the active surface of a soft plate allowing for the effective material removal and a substantial improvement of the surface finish of Al_2_O_3_ ceramic samples. The assessment of statistically significant differences between the means of the technological effects confirmed the stable course of lapping. Obtained results were compared to the results from lapping using other abrasive tools. It was demonstrated that selective laser sintering can be utilized to fabricate tools for the cheap, effective and environment-friendly abrasive finishing processing with minimum quantity abrasive dosing (MQAD).

## Introduction

The continuous development of grinding is towards an increased material removal rate (MRR) and at the same time towards an improved surface finish^1^. Lapping with the use of loose abrasives is one of the basic finishing processes for obtaining fine surface finish which is produced by a relatively low efficiency^2^. On the other hand, grinding with lapping kinematics integrates the advantages of both processes, i.e., high efficiency of grinding^3^ and good accuracy of lapping^4,5^. In general, finishing processes tend to be time-consuming and energy-intensive, requiring expensive tools and resources. It is possible to successfully reduce machining times while ensuring a clean process and energy efficiency, as presented on the example of hand polishing replaced by abrasive flow machining with a movable mandrel (AFMmm)^6^. Minimizing the quantity of the lubrication and abrasive slurry in finishing processes shows many technical and economic advantages^7,8^. Further development of abrasive processes such as grinding, lapping and polishing can be achieved through the use of additive technologies emerging as an environmentally friendly green manufacturing technologies which brings great benefits, including energy saving, reduced material consumption and more efficient production^9^.

Nowadays, many end-use parts for multiple industrial applications can be manufactured by additive technologies using different materials and composites^10^. The available 3D printing methods are differentiated in terms of how the physical object is built. There are several groups of AM methods, such as: powder bed fusion, material extrusion, vat polymerization as well as material and binder jetting^11^.

Difficulties associated with machining of difficult-to-cut materials, including hard and brittle ceramics characterised by porous structure, influencing the mechanical properties of all materials^12^ make it necessary to develop innovative tools allowing for cheaper and more efficient manufacturing. Chemical mechanical polishing (CMP) uses similar kinematical configuration as lapping and can also produce surfaces with fine surface finish and without subsurface damage to hard materials, such as silicon carbide^13^. Based on the sol-gel technology, Luo et al.^14^ and Lu et al.^15^ proposed a semi-fixed abrasive tool with diamond and alumina grains for SiC and sapphire substrates polishing. In general, fixed diamond abrasive polishing film enabled to obtain higher material removal rate *MRR*, while semi-fixed abrasive polishing film a lower surface roughness Ra parameter. Pyun et al.^16^ tried to combine the two-body and three-body abrasion modes during sapphire machining using a developed high-performance Cu-resin lapping plate. The application of relatively soft metal-resin tool with diamond slurry determined to transform abrasion mechanism. Consequently, compared to conventional metal plate, higher material removal and better surface quality were obtained.

Additively fabricated tools can be also successfully used in different abrasive processes as shown in^17^. These types of prototype tools can be categorised into three main groups, such as: metal-bonded, resin-bonded and polyamide powder-bed fusion abrasive tools. Incremental technologies provide many benefits in the fabrication of the new constructions and designs of tools used in grinding and lapping processes. For example, LPBF – laser powder bed fusion processes allow to produce tools with advanced and complex geometries, including strictly defined inner structures and shapes, such as pores, cooling channels or holes. Compared to conventional fabrication methods, additively fabricated tools from powders or resins are a cost-effective solution for small or unit batch production, while the production process is simpler and requires a smaller number of technological operations and devices. For instance, Denkena et al.^18^ used the laser powder bed fusion (LPBF) technique to fabricate NiTi diamond composites suitable for grinding tools to grind tungsten carbides. The diamonds grains were firmly held in the bond but the LPBF process parameters for pure metals were not completely transferable to metal diamond composites. Abrasive-mixed resindiamond bond grinding wheels produced by Stereolithography (SLA)-based method were investigated in^19,20^. In recent years, one of the most promising additive manufacturing techniques for industrial applications is selective laser sintering (SLS). In SLS process, a laser as a power source selectively sinters the particles of a polymer powder, fusing them together and building a physical object layer by layer. Moreover, Hon and Gil^21^ produced silicon carbide/polyamide matrix composites for the grinding wheels using SLS fabricating process but the effect of the process parameters and their interactions on mechanical and physical properties were not satisfactorily examined. Williams^22^ analysed the impact of a layer thickness of an abrasive mixed resin on the performance of lapping plates manufactured by SLA method. SLS method was successfully used for fabricating prototype grinding wheels with strictly defined internal cooling holes^23^.

The knowledge on the optimal process parameters for the fabrication of polyamide, resin or metal matrix composites along with Design for Additive Manufacturing (DfAM) tools and techniques can be a key issue for wider implementation of AM in the production of tool used in abrasive processes^24,25^. The successful application of additive processes for abrasive tools is limited by the effective control of their porosity and micro-structure^26^, but It is obviously accepted that additive technologies offer new capabilities to produce functional tools for abrasive processes.

The paper presents a prototype polyamide tool made by the SLS process for the single-sided lapping. In general, the active surface of a soft tool is more effectively embedded by loose diamond grains. Consequently, higher efficiency and improved surface finish of Al_2_O_3_ ceramic samples was achieved. Diamond grains were dosed only at the beginning of the process in a very small amount in comparison to typical free abrasive machining (FAM). In addition, shorter time and lower cost of the finish process were documented in relation to a conventional cast iron lapping plate. Obtained results of technological effects are compared to the results obtained in lapping using conventional and non-conventional abrasive tools. The results presented in the paper demonstrate that selective laser sintering can be utilized to fabricate tools for the cheap, effective and environment-friendly abrasive finishing processing with minimum quantity abrasive dosing - MQAD. The term MQAD is proposed by authors of this paper for the first time and is based on the main novelty of the given solution over standard slurry-based lapping which is the single dosing of a very small amount of abrasive past (4 ml) and D107 diamond grains (4.4 g) only once before 120 min of processing. In the available literature there is no information about such a small dosage of abrasive grains effectively used for such a long time on a working surface of an additively fabricated tool.

## Materials and methods

### The lapping tool

**Fig. 1:**
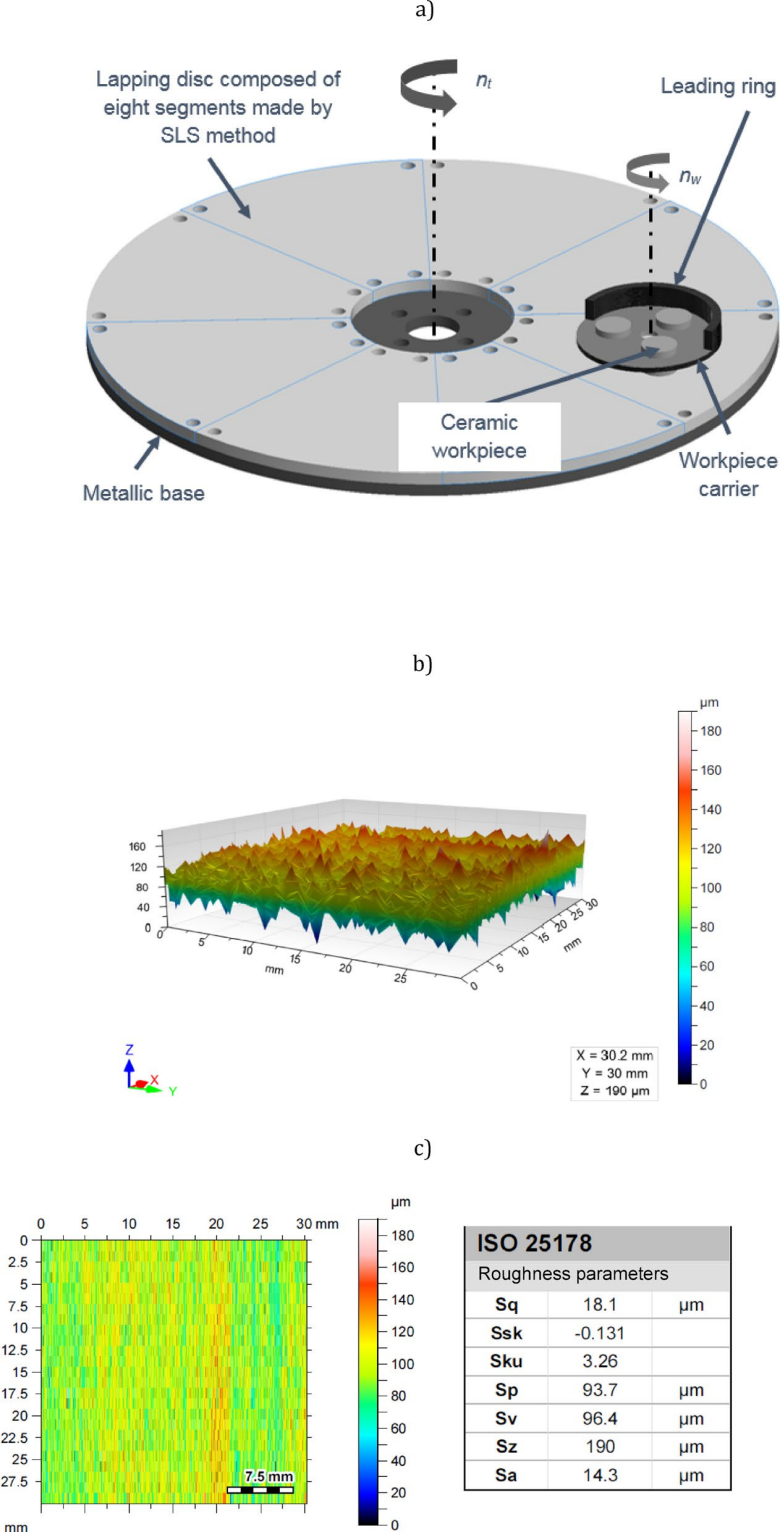
SLS-fabricated lapping plate: (**a**) kinematical configuration of the single-sided lapping process, (**b**) an exemplary surface texture of the active tool surface and (**c**) height and amplitude roughness parameters; where *n*_*t*_ – rotational speed of a lapping plate and *n*_*w*_ – rotational speed of a leading ring.

The uniform flat surface of the lapping plate presented in Fig. 1 was obtained by screwing eight segments printed by SLS technology on EOS Formiga P100 3D printer to the metal body, with the unit production cost of about 200 USD. The estimated cost of fabrication of the abrasive segments includes the cost of the PA2200 material used, the cost of performing the process of building the tool on the 3D printer as well as basic post-production treatment (post-processing) associated with cleaning the segments from the polyamide powder after the process. The segments were made of the polyamide PA2200 powder characterised by the properties shown in Table 1, using the process parameters specified in Table 2. Printed elements were sandblasted and, as a result, unsintered powder particles were removed by compressed air. The surface topography of 8 fabricated segments presented in Fig. 1b was measured using the Hommel Etamic T8000 tester. The irregular structure obtained with the average value of the Sa surface roughness parameter 15 μm – Fig. 1c, is characteristic for the parts produced by the SLS process. No additional post-production treatment (post-processing) of the active surface of the tool was performed.

**Table 1:** Selected mechanical and physical properties of the polyamide PA2200^27^.

Property	Value
Mechanical properties	
Shore D hardness (15 s)	75
Tensile modulus	1650 MPa (X, Y, Z direction)
Tensile strength	48 MPa (X, Y direction)
Tensile strength	42 MPa (Z direction)
Flexural Modulus (23 °C)	1500 MPa (X direction)
Strain at break	18% (X, Y direction)
Strain at break	4% (Z direction)
**Thermal properties**	
Melting temperature (20 °C/min)	176 °C
Vicat softening temperature (50 °C/h 50 N)	163 °C
**Other properties**	
Density after laser sintering	930 kg/m^3^
Powder colour	white

**Table 2:** Parameters of the 3D printing process carried out on a EOS Formiga P100 3D printer using a powder material of PA2200 grade.

SLS process parameter	Value
Thickness of a single layer	100 μm
Laser type	CO_2_
Power of laser	30 W
Length of a laser beam	10.2–10.8 μm
Printing speed	10 mm/s
Printing resolution	0.005 mm

### Experimental setup

In experimental tests, cylindrical samples made of Al_2_O_3_ ceramic material with an outer diameter *d*_*w*_ = 34 mm and an initial height *h*_*w*0_ = 30 mm, were machined. After each test, the sample height as well as the roughness and waviness of machined surfaces were measured at three selected points. The height measurement was conducted using a Mitutoyo micrometer with a resolution of 1 μm, while the roughness and waviness of machined surfaces were measured using a HOMMEL TESTER T500 contact profiler according to DIN4777 standards.

**Fig. 2:**
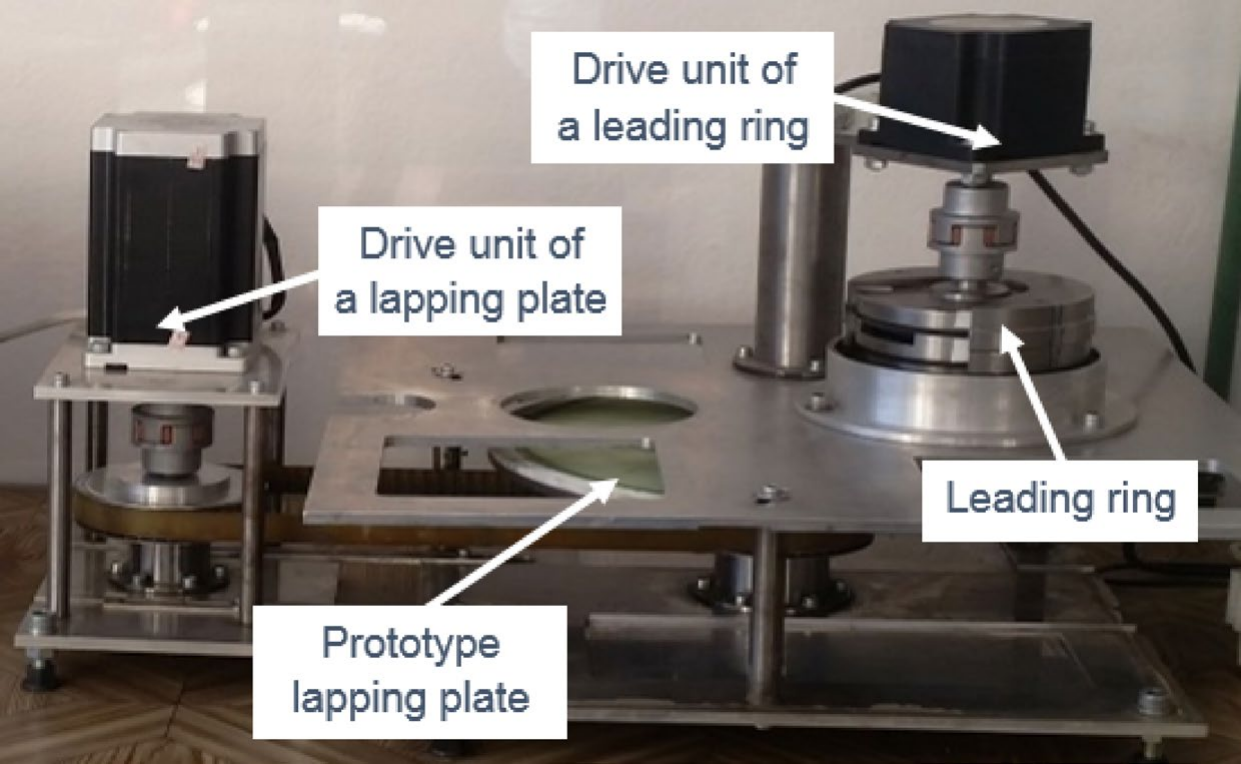
Experimental lapping setup using composed lapping tool.

**Fig. 3:**
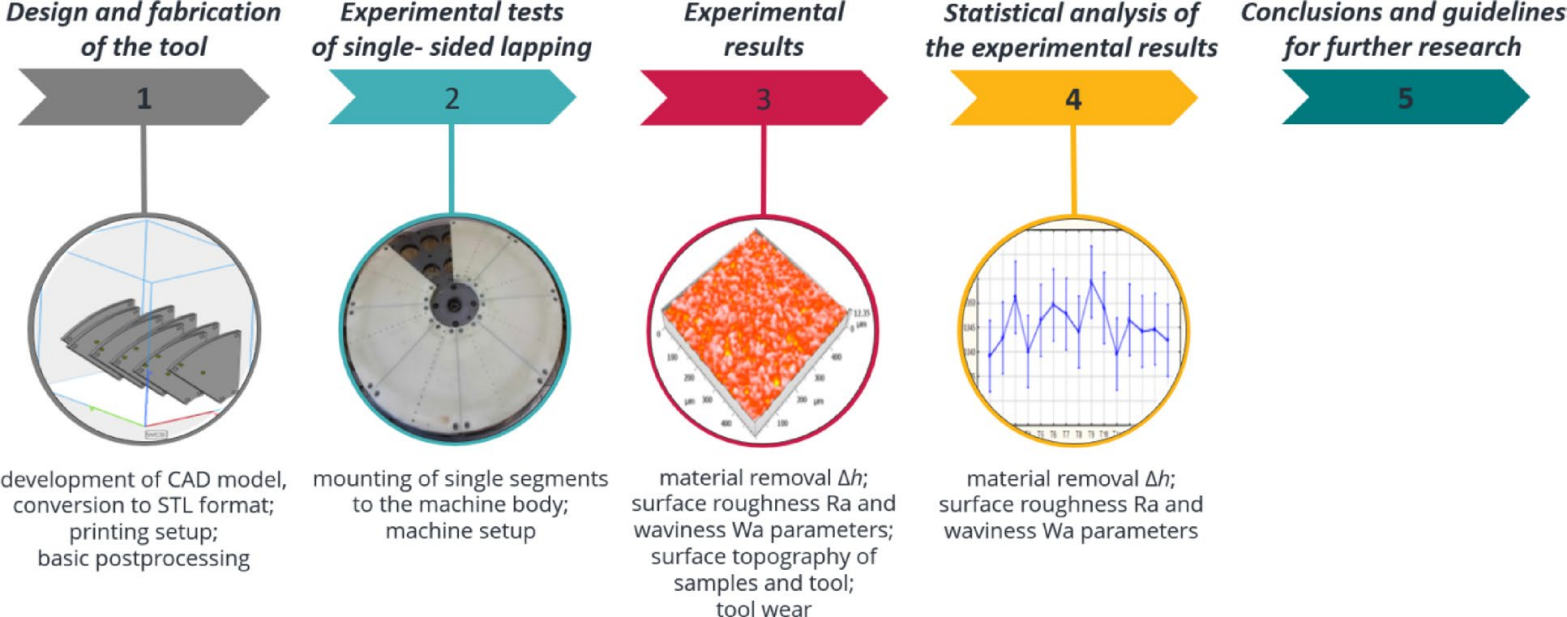
Flowchart of the experimental procedure.

The process parameters used in all tests are specified in Table 3. Lapping experiments were carried out on the test stand for single-sided lapping or lap-grinding^5^, with two independent and programmable drives of the lapping plate and the leading ring shown in Fig. 2. The required unit pressure was controlled by means of metallic weights placed inside the leading ring. Only machined surfaces were in contact with the tool’s active surface through the abrasive slurry, with no contact between the leading ring and the lap. The experimental procedure was demonstrated in Fig. 3.

**Table 3:** Lapping conditions kept during experiments for all tests T1-T30.

Lapping disc	
Outer tool diameter	*d*_*o*_ = 380 mm
Inner tool diameter	*d*_*i*_ = 90 mm
Material of the working elements	PA2200
**Lapping parameters**	
Rotational speed of a lapping plate	*n*_*t*_ = 120 min^−1^
Rotational speed of a leading ring	*n*_*w*_ = 60 min^−1^
Unit pressure	*p* = 12 kPa
Duration of a single test	*t*_*t*_ = 4 min
**Abrasive suspension**	
*Abrasive paste*	SD 28/20
Grains in abrasive paste	Diamond D28
Volumetric grain concentration in abrasive paste	20%
Volume of abrasive paste	4 ml
*Loose abrasive grains*	Diamond D107
Volume (mass) of loose grains	2.5 ml (4,4 g)
*Lubricant*	Machining oil
Lubricant flow rate	*Q*_*lub*_ = 0.5 ml/min
**Workpiece**	
Material	Al_2_O_3_ ceramic
Vickers hardness HV10	1100 MPa
Diameter	*d*_*w*_ = 34 mm
Initial height	*h*_*w*0_ = 30 mm

## Experimental results

It was preliminarily assumed that abrasive grains of D28 grade are sufficient for abrasive finishing of Al_2_O_3_-based ceramics, but due to a very small material removal rate larger D107 diamond grains were additionally supplied into the working zone. The use of only abrasive paste without additional loose and large diamond grains did not enable the cutting of hard ceramics. Most probably, the elastic tool with porous structure did not allow for sufficient protruding of D28 abrasive grains above the active tool surface, so that the workpiece material could be effectively removed. In consequence, a dual-phase lapping process was performed. First, basic material allowance was removed by bigger D107 abrasive grains whereas the residual surface irregularities were smoothed by a mixture of diamond grits with small D28 and large sizes D107, which were crushed into smaller pieces during machining. Some previous experimental results of a novel lapping process were reported in Ref^28,29^.

The abrasive paste SD 28/20 of 4 ml volume and loose diamond grits D107 (2.5 mL) were evenly distributed on the tool’s active surface only once before the whole test lasting 120 min. The machining oil was used as a lubricant with the low flow rate of *Q*_*lub*_ = 0.5 ml/min. The linear material removal rate as well as the roughness and waviness parameters of the machined surfaces, were measured after 4 min lapping tests.

It is important to note that the sample height was reduced from the initial height of 30_−0.025_ mm to the final height of 28.6_±0.005_ mm in order to remove surface irregularities and defects produced in the initial operation. After the long-time test of 120 min, a microscopic analysis of the selected sections of the tool’s active surface was performed and concurrently the linear tool wear was measured.

**Fig. 4:**
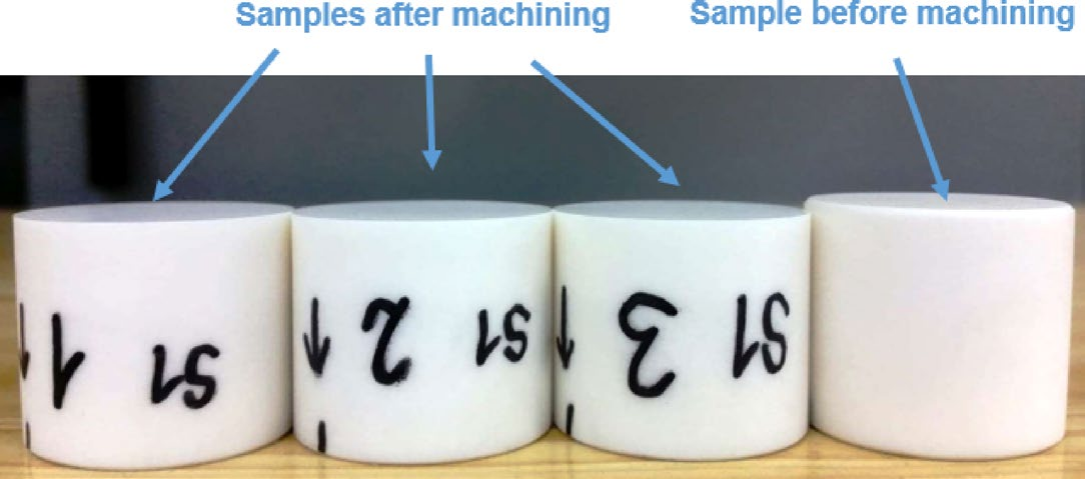
The differences in the height of Al_2_O_3_ ceramic samples before and after 120 min of machining using a prototype tool made by the SLS method.

**Fig. 5:**
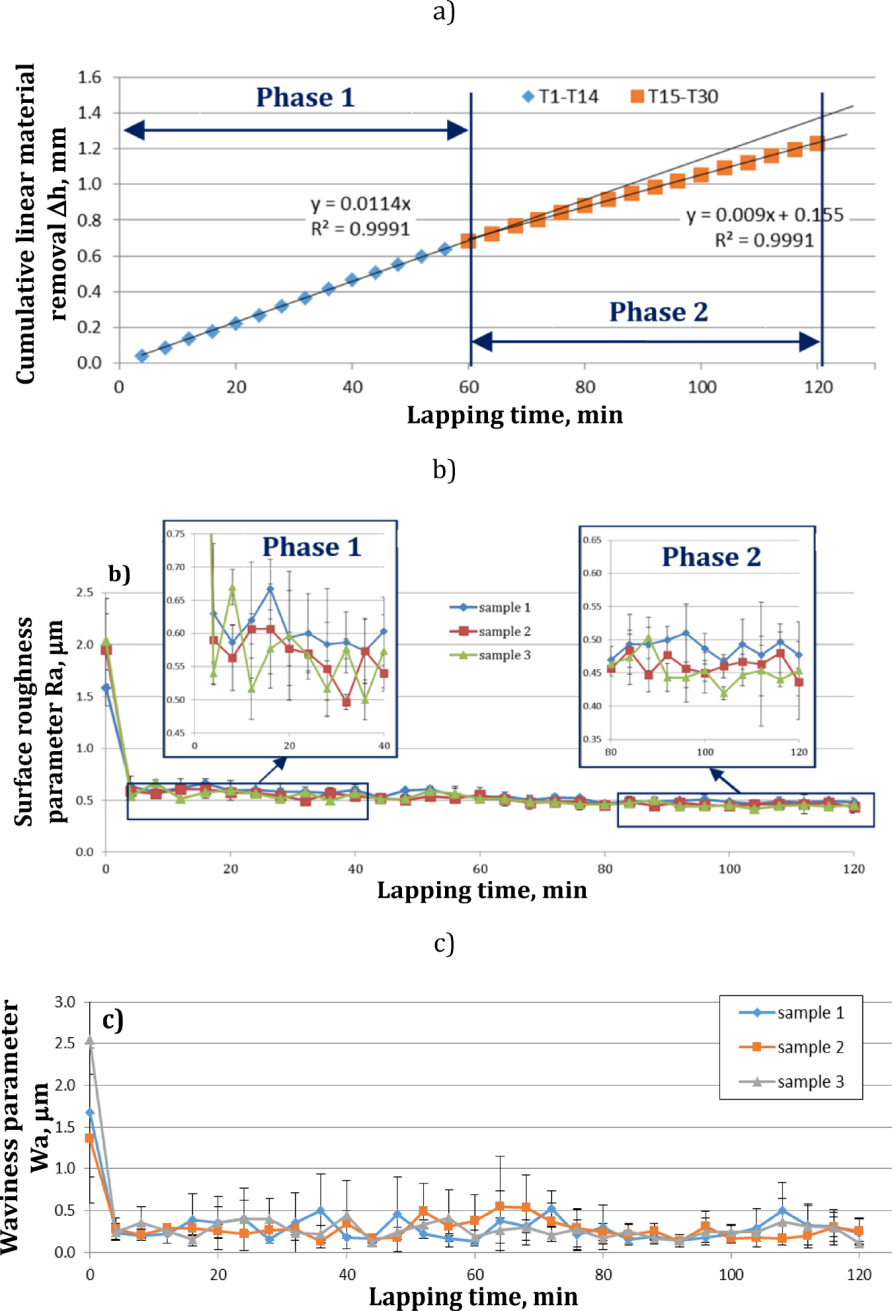
Results obtained for single-sided lapping of Al_2_O_3_: (**a**) cumulative linear material removal (Δ*h*), (**b**) surface roughness parameter (Ra), (**c**) waviness parameter (Wa); error bars show (±) standard deviation.

As can be seen in Figs. 4 and 5, the conducted experiments resulted in more effective material removal as well as a significant reduction of the surface roughness and waviness parameters. After the first 4 min of lapping, a large decrease in roughness and waviness parameters was observed. After subsequent tests, the roughness and waviness parameters remained at a similar level (see Fig. 5b and c). The diamond grits embedded in the soft material perform two-body abrasion process. Compared to the conventional lapping which is modelled as the three-body abrasion^2^, the ceramic material is more efficiently removed during a combined 2-body and 3-body abrasive wear^5^.

As can be seen in Fig. 5a, the material removal rate is nearly linear, especially when the test time is divided into two phases. In particular, after 60 min of lapping, a slighter decrease in the material removal rate was observed. This effect probably results from the partial dulling of the edges of diamond grains. Simultaneously, the dulling of the cutting edges causes that the roughness of machined surfaces after 60 min of lapping is slightly reduced (see Fig. 5b).

**Fig. 6:**
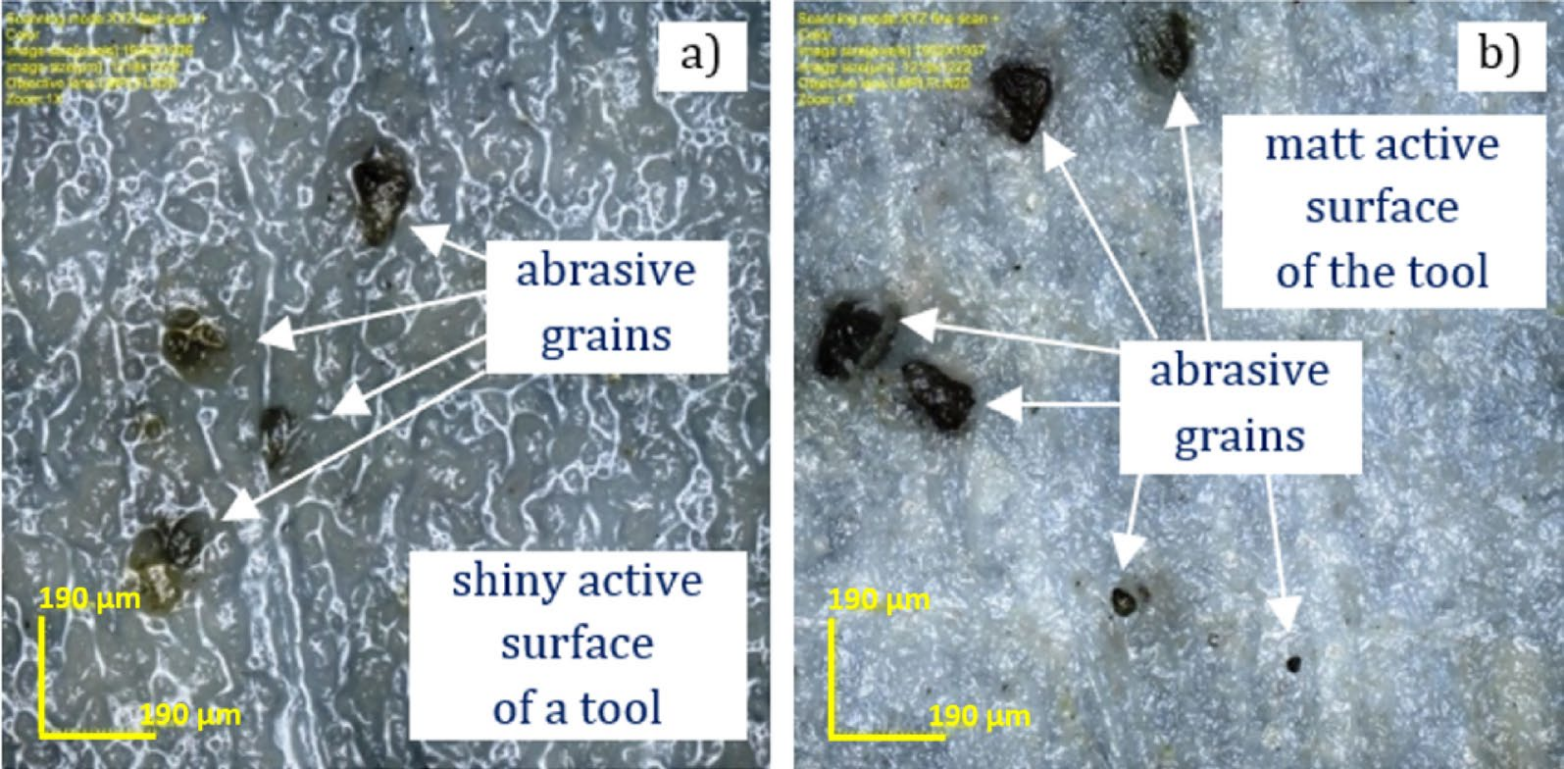
Selected sections of the tool’s active surface after 120 min of abrasion: (**a**) before and (**b**) after cleaning from the residual abrasive suspension.

Before the tests, it was expected that the time of effective cutting would be much shorter than it was achieved. Surprisingly, the lapping process was conducted effectively for 120 min only with a single supply of abrasive grains before the first test. Moreover, further cutting was feasible even after the last thirtieth test (after 120 min). Interruption of the experiments allowed the characterization of the state of the active tool surface after 120 min of abrasion following the previously described procedure. The analysis of surface images obtained using a confocal microscope, model OLYMPUS LEXT OLS4000 and Sensofar S-Neox optical profilometer confirmed the presence of undamaged abrasive grains and their fragments embedded in the lapping disc (see Fig. 6a). The removal of the residual abrasive suspension from the lapping disc also reveals continuous embedding of new diamond grains (see Fig. 6b). During lapping, the abrasive grains were embedded in a soft and elastic material, enabling effective removal of the hard and brittle ceramic material. Large diamond grains D107 trapped between the lapping plate and the workpiece caused higher material removal rates. In addition, they were also crushed into smaller pieces which increased the grain concentration in some areas of the active tool surface. Continuous embedding of diamond grains and the resulting continuous material removal demonstrate a predominant property of the SLS printed tool over resin-based abrasive tools used in other studies^19,20^.

**Fig. 7:**
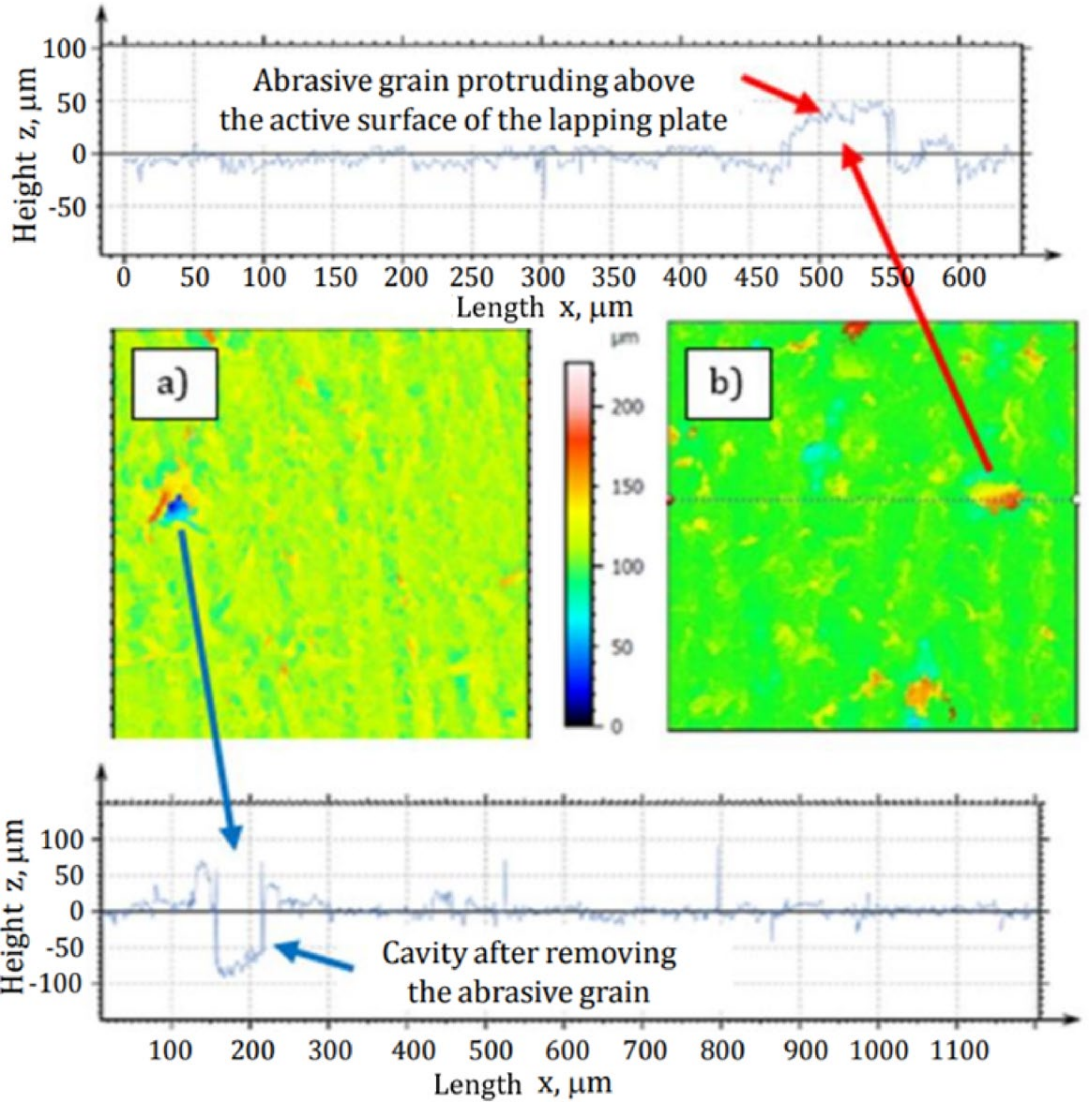
Contour maps of the active surface of the prototype lapping disc together with selected cross-sections along the profiles from the area with: (**a**) a removed and (**b**) embedded diamond grain.

The analysis of cross-sections obtained by confocal microscopy also confirmed the presence of diamond grains embedded in the lapping disc. It enables the approximate estimation of their exposure above and below their penetration into the active surface of the tool as shown in Fig. 7.

Due to the long-lasting machining, the previously embedded diamond grains were also removed as documented by appropriate discontinuities within the surface profile presented in Fig. 7a. The contour maps and the shape of profiles indicate a deep embedding and penetration of diamond grains (~ 80 μm) in the active surface of the elastic tool with a characteristic porous structure. The removal of abrasive grains from the lapping disc as well as their crushing into smaller pieces, in addition to the blunting of the cutting edges, reduced the lapping performance by approx. 8% after 60 min of machining time (see Fig. 5a).

Another effect of the printed tool was a significant reduction of the roughness of the ceramic workpieces despite the initially high value of the Ra parameter of 1.5–2 μm (see Fig. 5b). The primary advantage of the proposed solution over standard slurry-based lapping is the single dosing of a very small amount of diamond abrasive grains during 120 min of the process. It causes that the tested abrasive process is more economical and environment-friendly. The higher process efficiency in comparison to conventional lapping suggests the process transition from three-body to two-body abrasion. At the initial stage of processing, loose grains were able to rotate between the working surface of the tool and the machined surface. Due to this, three bodies were involved in the process, namely grains, tool and the workpiece. During the process, more and more loose grains were fixed in the lapping tool to form a single tool, similar to a grinding tool. Thus, the application of SLS printed tool with D107 diamond grains allows performing more efficient abrasion, similar to grinding or lap-grinding^2,5^.

**Fig. 8:**
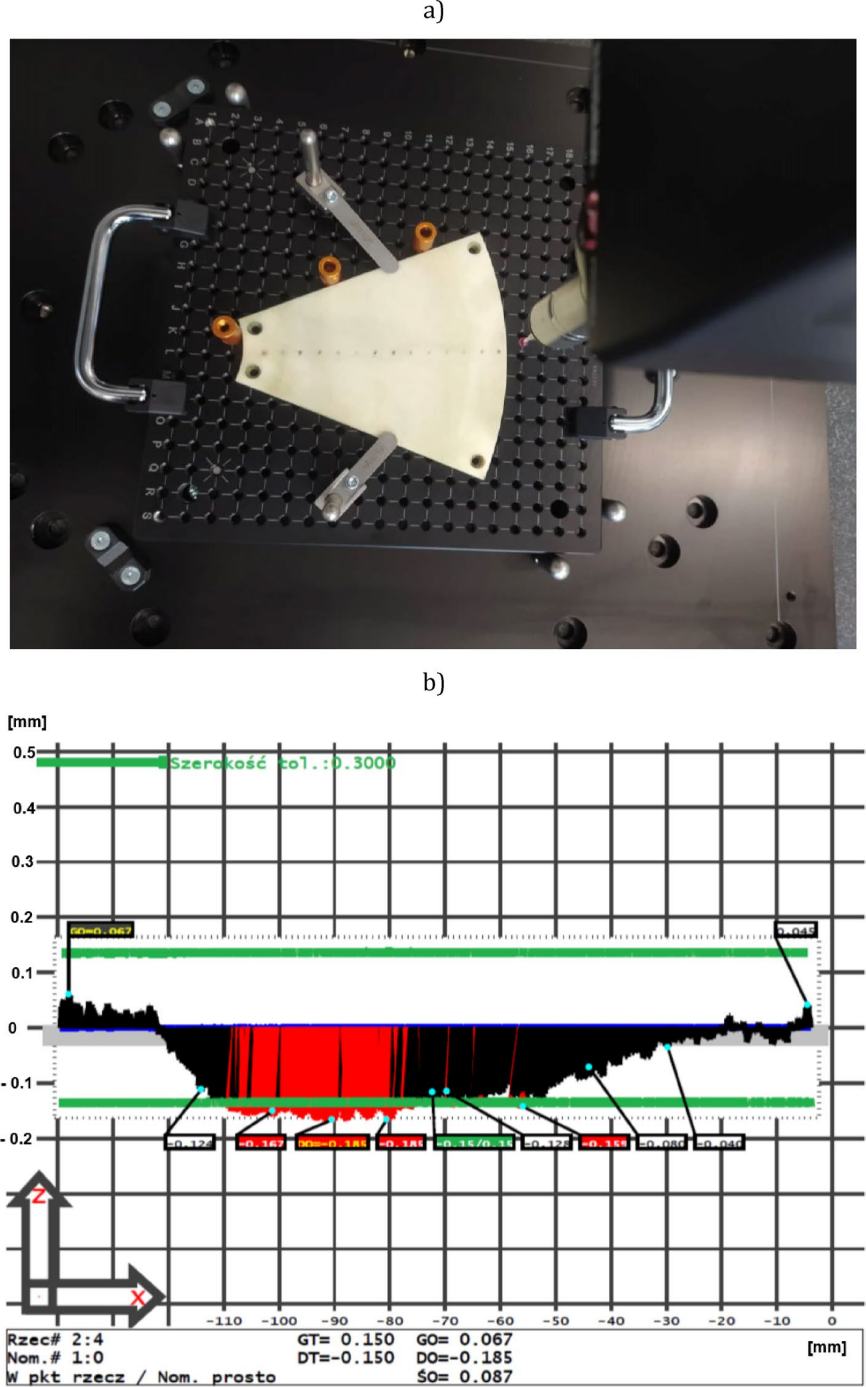
Measurement of a profile of a single abrasive segment on a MiSTAR 555 CNC coordinate measuring machine: (a) general arrangement, (b) an exemplary profile after machining measured along the centreline.

Another important aspect of all flattening processes with lapping kinematics is the substantial reduction of tool wear. Similarly, the use of a SLS printed tool is distinguished not only by high efficiency but also by a relatively low wear. The visual inspection performed during and after the lapping process did not indicate excessive tool wear. In order to quantify tool wear, the profile of an abrasive segment was measured along its centreline using a MiSTAR 555 CNC coordinate measuring machine – Fig. 8. The measured profile presented in Fig. 8b confirmed a relatively low wear of the additively composed tool, much smaller than for the resin lapping discs^19,20^ and at the similar level as for cast iron lapping plates^2^. The better abrasive capability of the tool active surface, in contrast to both resin-based and electroplated abrasive tools, causes that the lapping process of hard technical ceramics can be continued after assumed 120 min.

**Fig. 9:**
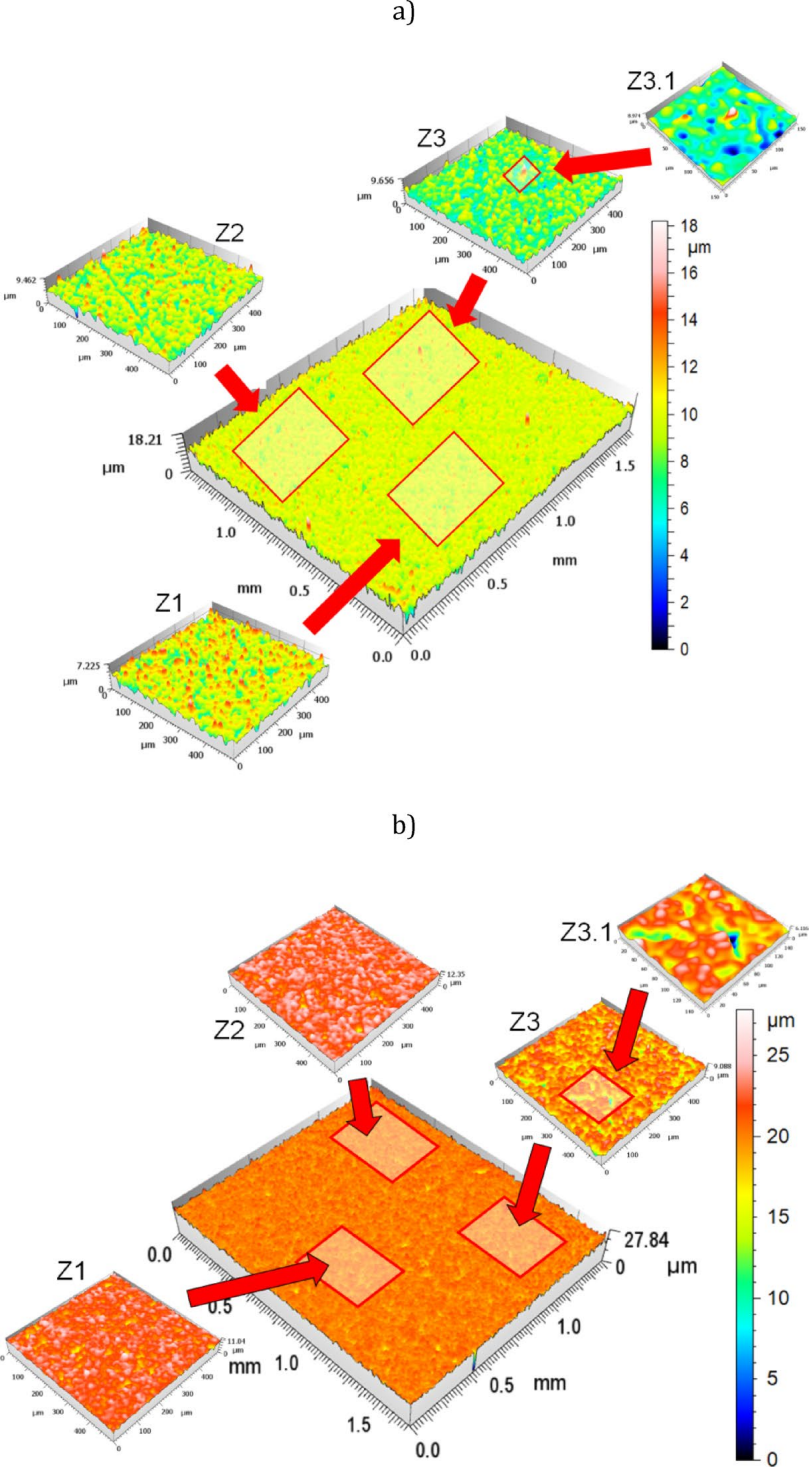
Exemplarily surface topographies with selected magnified fragments of: (a) initial and (b) lapped surfaces.

Figure 9a and b illustrate a comparative analysis of the surface topographies of the initial (Fig. 9a) and lapped (machined) surfaces (Fig. 9b), each with dimensions of 1.5 mm × 1.5 mm. From each surface, three sub-images labeled Z1, Z2, and Z3 were extracted, representing areas of 500 μm × 500 μm. Furthermore, a nano-scale region with dimensions of 150 μm × 150 μm was isolated from image Z3 and denoted as Z3.1. This methodological approach to surface topography mapping enables a precise differentiation of roughness variations within the analysed area and facilitates a comprehensive assessment of surface homogeneity.

In this study, both the visualization of surface topographies through isometric imaging and the quantification of fundamental 3D roughness parameters were performed using commercial SensoMAP Premium v 8 software. The obtained results showed that the Sa parameter values for the initial and lapped surfaces were approximately (1.1–1.2) µm and 0.5 μm, respectively. Notably, the measurement error for the final ceramic sample surfaces did not exceed 0.05 μm. At the nano-scale, the presence of minor defects, such as extremely small voids and embedded nanodiamond grains, was detected within the Z3.1 area (Fig. 9a and b). For a more detailed characterization of surface topographies produced by cutting and abrasive processes, readers are referred to **Ref.**^30^.

In the case of functional surfaces with predominant load-bearing capabilities, the primary factor is the main heredity index, which is defined by the relationship between skewness (Rsk) and kurtosis (Rku) within the Rku–Rsk envelope (equivalently, Sku–Ssk)^31^. It is important to highlight that the Ssk (Rsk) parameter provides valuable insights into surface characteristics relevant to various wear types and operational conditions, while the Sku (Rku) parameter is used to identify the presence of peak or valley defects on the surface.

As demonstrated in this study, the lapping process produced surfaces characterized by negative Ssk values and high positive Sku values. Specifically, the Sku values for the three analyzed surface areas (Z1, Z2, and Z3), shown in Fig. 9b, were 9.73, 6.35, and 14.9, respectively, while their corresponding Ssk values were − 0.955, −0.847, and − 1.66. It should be noted that the Sku and Ssk values after lapping were higher than those obtained for the initial surfaces (Sku = 3.1, 5.19, 3.53; Ssk = −0.34, −0.83, −0.48 for Z1, Z2, and Z3, as presented in Fig. 9a). Although the lapping process resulted in an increase in these parameters, the surface character remained consistent with the initial topography, indicating that lapping preserved the overall amplitude distribution morphology while enhancing peak sharpness and valley depth.

## Statistical analysis of the experimental results

**Fig. 10:**
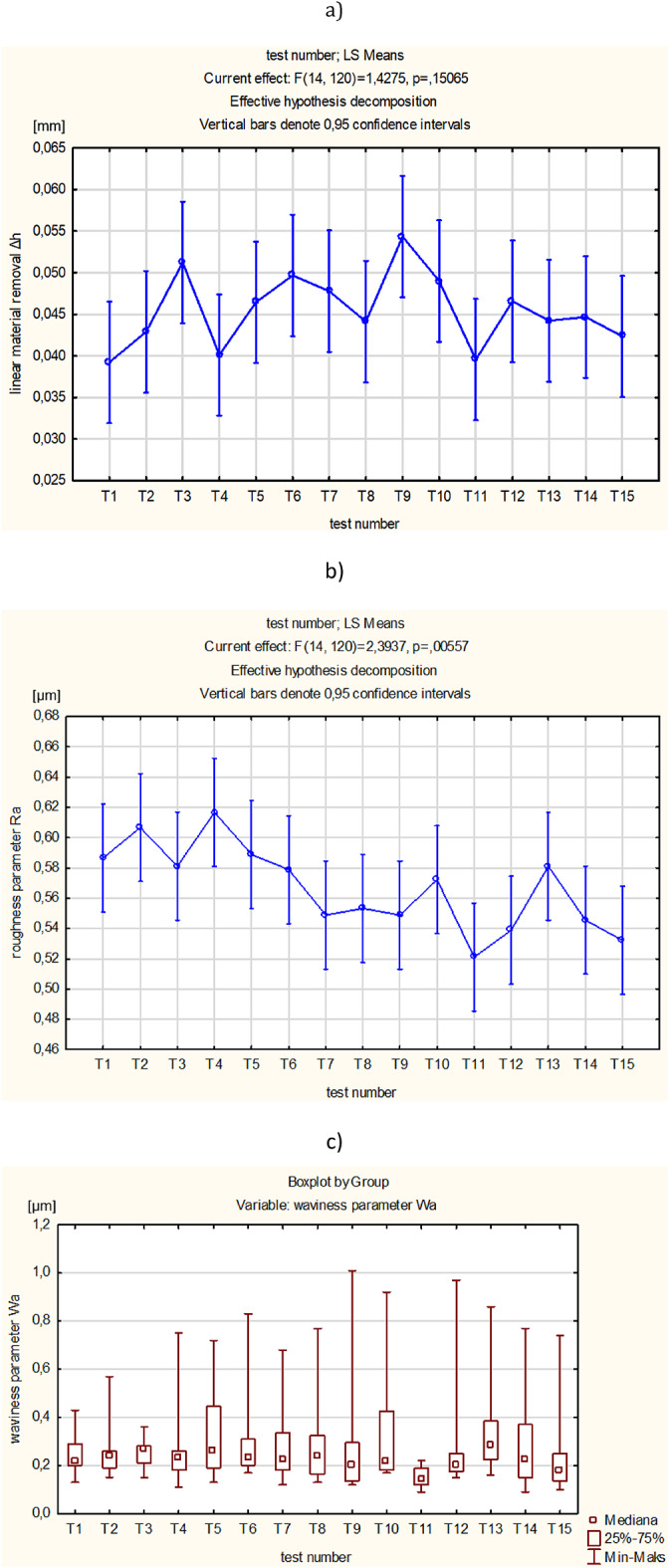
Graphical form of analysis of variance ANOVA between tests T1-T15 using: (**a**) LS Means for linear material removal Δh, (**b**) LS Means for roughness parameter Ra and (**c**) Boxplot for waviness parameter Wa.

**Fig. 11:**
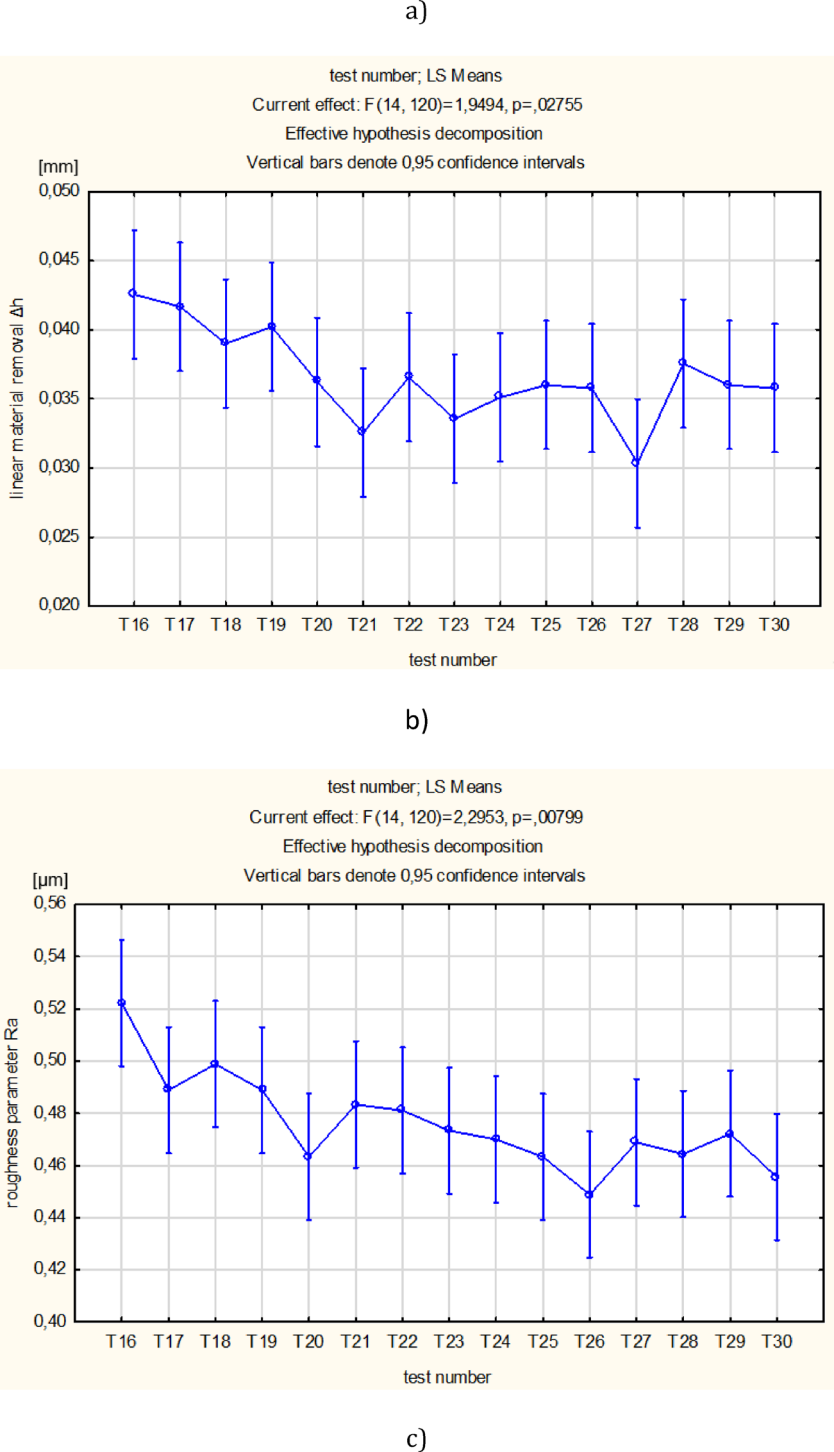

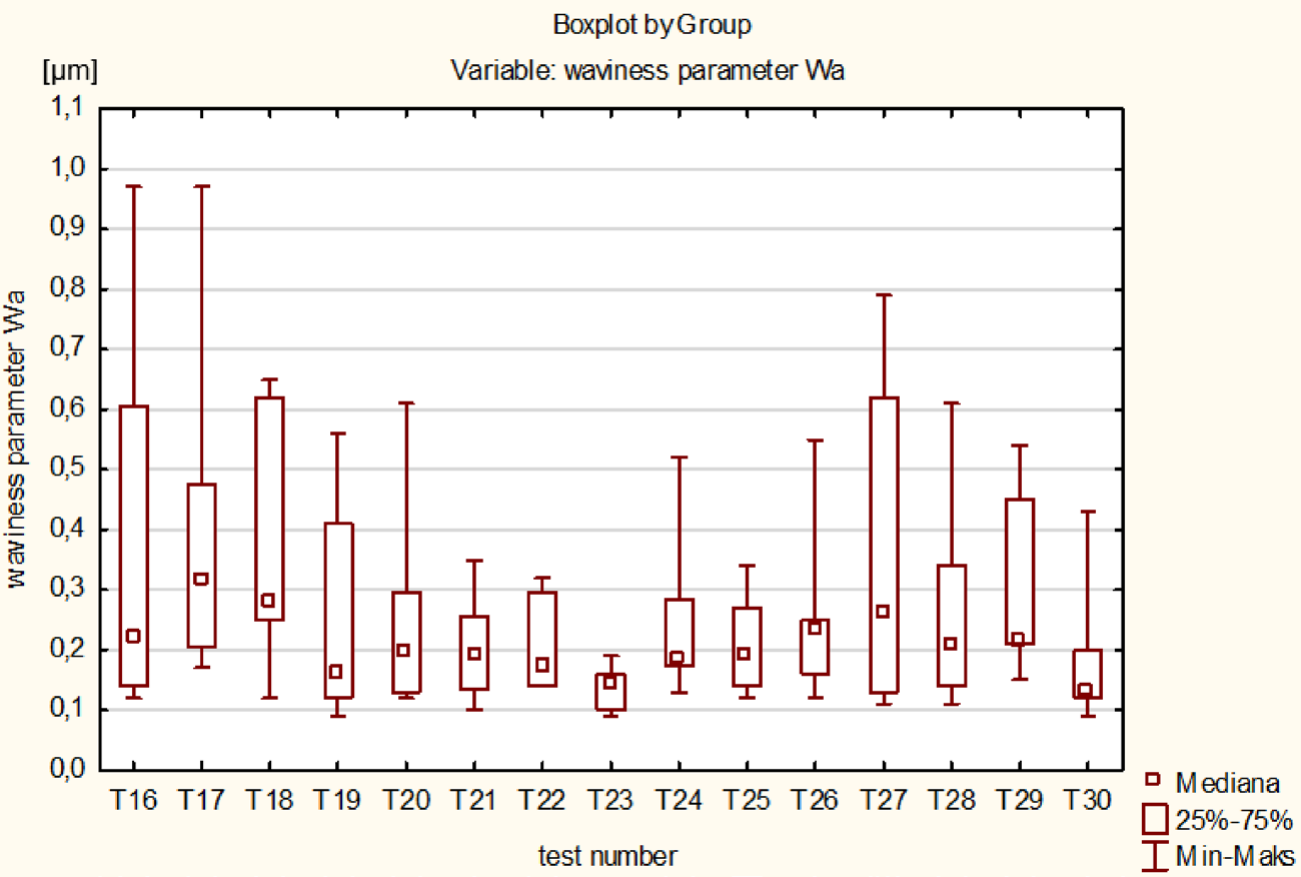
Graphical form of analysis of variance ANOVA between tests T16-T30 using: (**a**) LS Means for linear material removal Δh, (**b**) LS Means for roughness parameter Ra and (**c**) Boxplot for waviness parameter Wa.

Significant differences between the mean values of technological effects was tested by analysis of variance one-way ANOVA using Statistica software, for a significance level of α = 0.05. The statistically significant differences between the fifteen means of the T1-T15 tests for *t* ≤ 60 min (see Fig. 10) and fifteen means of the T16-T30 tests for *t* > 60 min (see Fig. 11) were verified. After assessing the normality of the data distribution using the Shapiro-Wilk test and the homogeneity of variance using the Levene’s test, the parametric ANOVA test was applied for the linear material removal Δ*h* and the roughness parameter Ra, and the non-parametric Kruskal-Wallis ANOVA & Median test for the waviness parameter Wa, respectively. Significant differences between the means (*p*-value < 0.05) occurred only for the roughness parameter Ra in both analysed processing periods and for the linear material removal Δ*h* for *t* > 60 min – Table 4. In order to indicate tests with significant differences between means, an additional post-hoc analysis was performed using the Tukey test. In the period *t* ≤ 60 min, significant differences appeared only for tests T4 and T11 for the parameter Ra, indicating a relatively stable course of machining. A greater number of significant differences occurred for *t* > 60 min and three times related to the T16 test. Due to the less stable process for *t* > 60 min, and because the T16 test was the first test in the Phase 2 of machining **(**Fig. 5**)**, the original distribution was changed to include the T16 test in the Phase 1. Thus, for the new distribution, the statistical significance of differences between the sixteen means from T1-T16 tests for *t* ≤ 64 min and between the fourteen means from T17-T30 tests for *t* > 64 min, was tested again – Table 5. Significant differences occurred twice, only for the roughness parameter Ra and for *t* ≤ 64, confirming the stable machining process in both newly adopted phases, the Phase 1 extended and the Phase 2 shortened by the duration of the T16 test. In conclusion, shifting the T16 test to phase 1, and thus determining new distributions for *t* ≤ 64 min and *t* > 64 min, resulted in a decrease in the number of significant differences between machining tests. Thus, the statistical analysis allowed a more adequate division of the processing into two phases corresponding to the obtained measurement data.

**Fig. 12:**
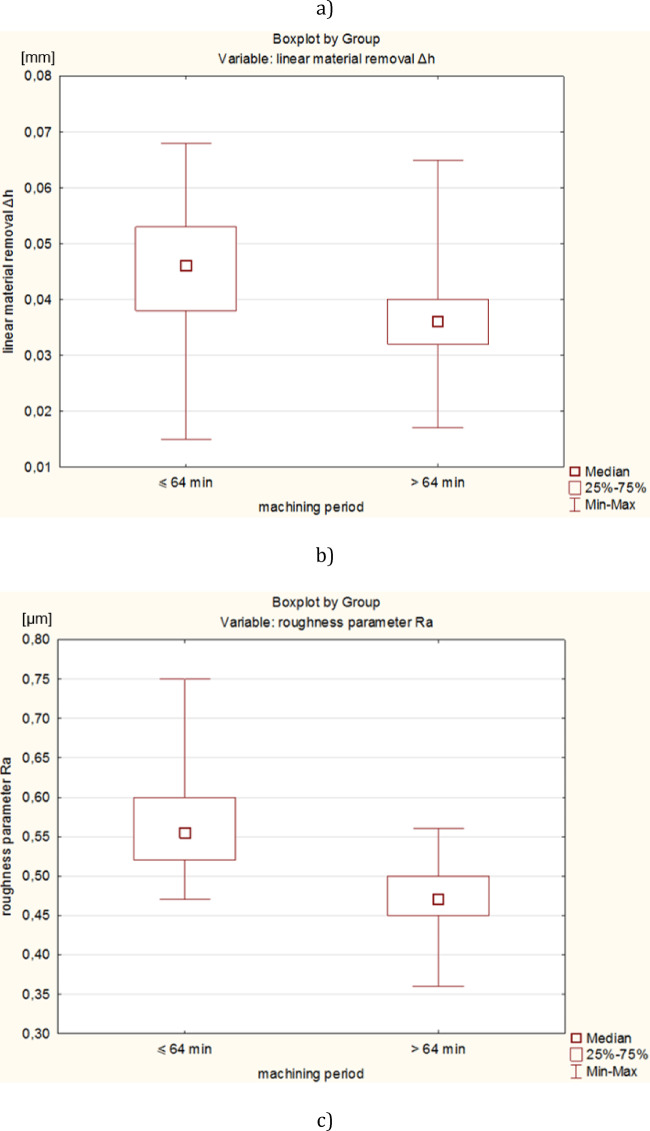

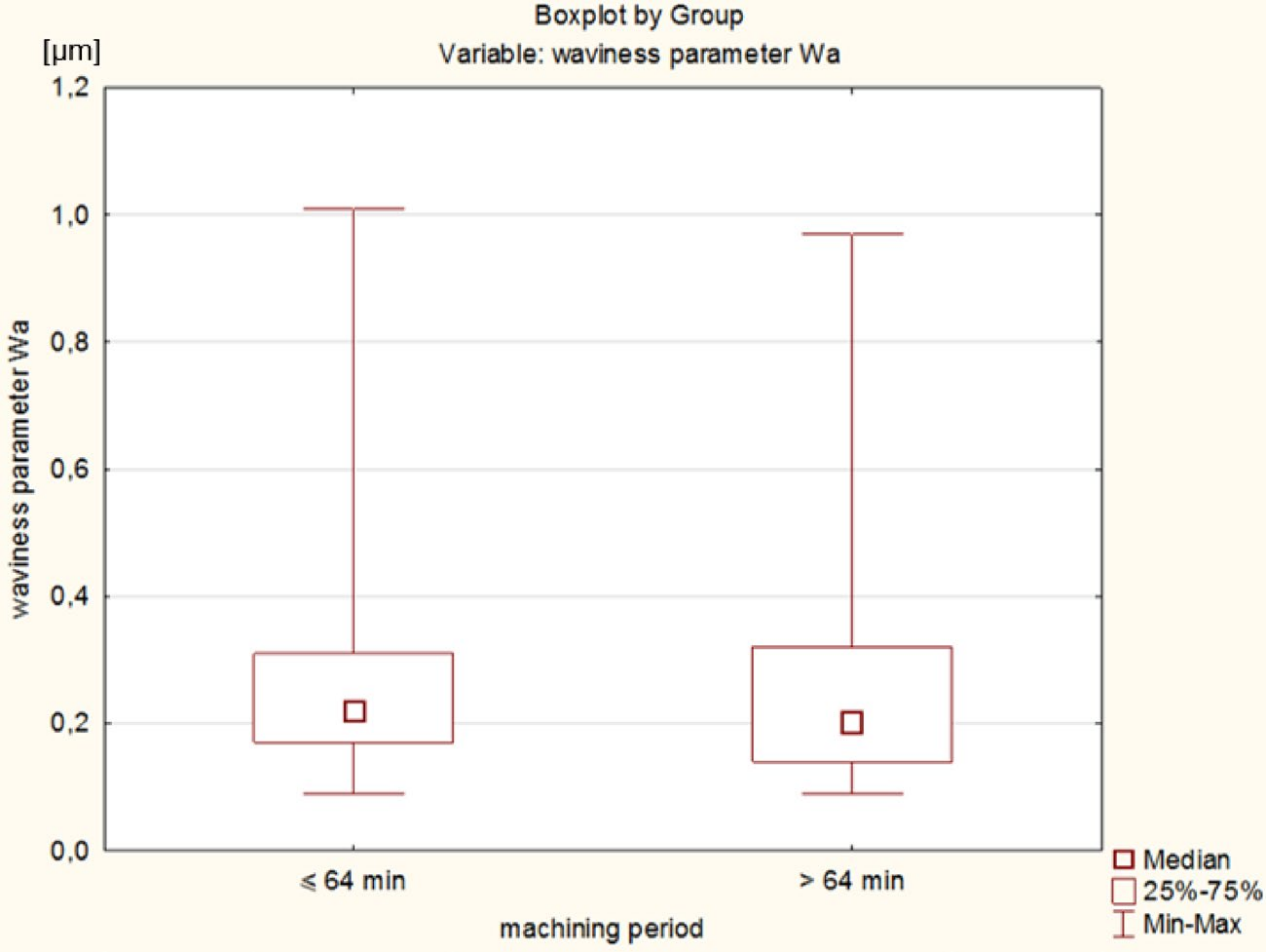
Graphical form of Kruskal-Wallis ANOVA & Median test using Boxplot for: (**a**) linear material removal Δh, (**b**) roughness parameter Ra and (**c**) waviness parameter Wa.

Finally, due to the small number of significant differences related only to the roughness parameter Ra, two means of all tests from the newly adopted machining phases were compared for *t* ≤ 64 min (one mean from all T1-T16 tests) and for *t* > 64 min (one mean from all T17-T30 tests) – (see Fig. 12). After assessing the normality of the data distribution using the Shapiro-Wilk test and the homogeneity of variance using the Levene’s test, the non-parametric Kruskal-Wallis ANOVA & Median test was used – Table 6. Significant differences between the means occurred for the linear material removal Δh and the roughness parameter Ra, which reflects the experimental results shown in Fig. 6. Significantly lower efficiency and roughness characterise the second machining phase (Phase 2), which is mainly associated with lowering the abrasive properties of the tool, while the waviness of the surface is not significantly different in both phases.

**Table 4:** Analysis of variance ANOVA between the fifteen means of the T1-T15 and T16-T30 tests.

T**echnological effect**	Source of variation (test numbers)	Machining period t	p-value	Statistical significance	Tests with significantly different means
linear material removal Δh	T1-T15	≤ 60 min	0,15065	no	-
roughness parameter Ra			0,00557*	yes	T4 and T11
waviness parameter Wa			0,3555	no	-
linear material removal Δh	T16-T30	> 60 min	0,02755*	yes	T16 and T27
roughness parameter Ra			0,00799*	yes	T16 and T26; T16 and T30
waviness parameter Wa			0,1220	no	-

**Table 5:** Analysis of variance ANOVA between the sixteen means of the T1-T16 tests and the fourteen means of the T17-T30 tests.

Technological effect	Source of variation (test numbers)	Machining period t	*p*-value	Statistical significance	Tests with significantly different means
linear material removal Δh	T1-T16	≤ 64 min	0,16695	no	-
roughness parameter Ra			0,00142*	yes	T4 and T11; T4 and T16
waviness parameter Wa			0,4657	no	-
linear material removal Δh	T17-T30	> 64 min	0,06603	no	-
roughness parameter Ra			0,20332	no	-
waviness parameter Wa			0,0843	no	-

* p-value indicating statistically significant differences (p-value < 0.05).

**Table 6:** Analysis of Kruskal-Wallis ANOVA & Median between two means of all tests for *t* ≤ 64 min and *t* > 64 min.

Analysed result	Source of variation (machining periods)	p-value	Statistical significance
linear material removal Δ*h*	*t* ≤ 64 min and *t* > 64 min	p ≪ 0,05*	yes
roughness parameter Ra		p ≪ 0,05*	yes
waviness parameter Wa		0.0793	no

* p-value indicating statistically significant differences (p-value < 0.05).

## Discussion

**Fig. 13:**
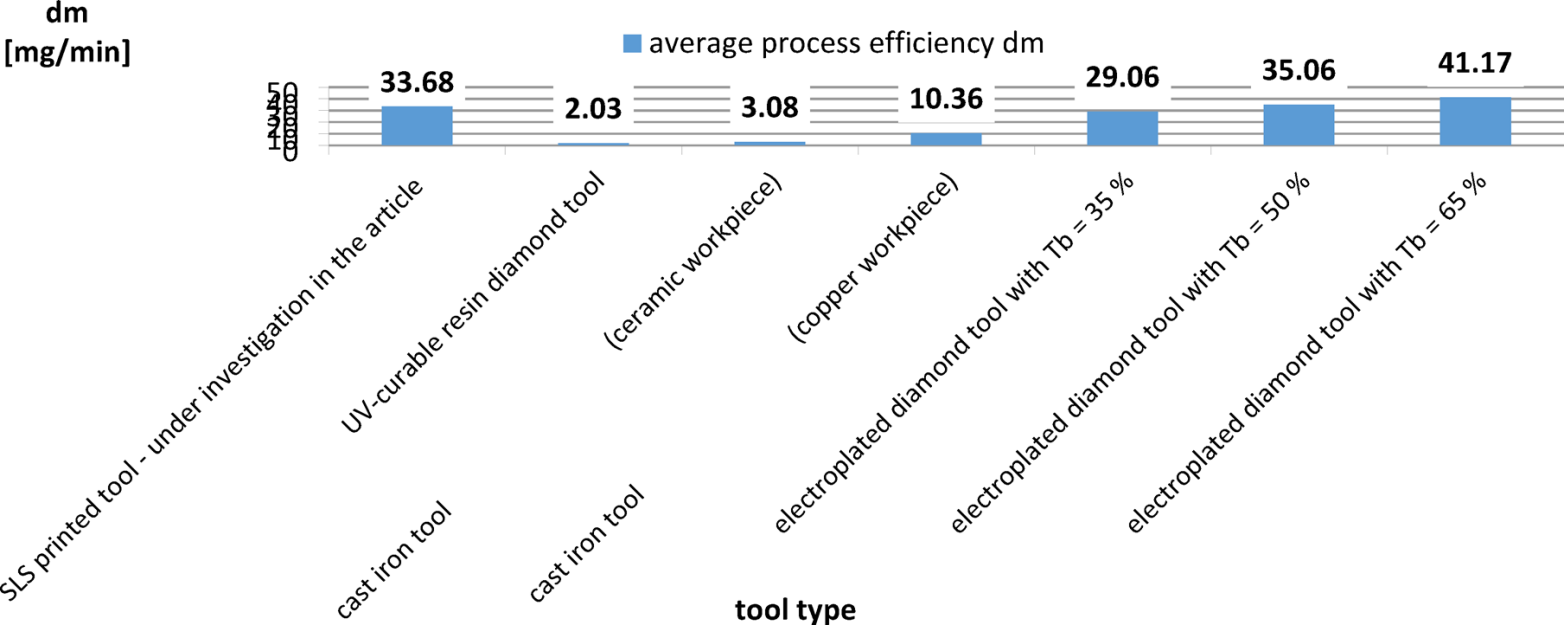
Process efficiency dm obtained in lapping using different tools.

**Fig. 14:**
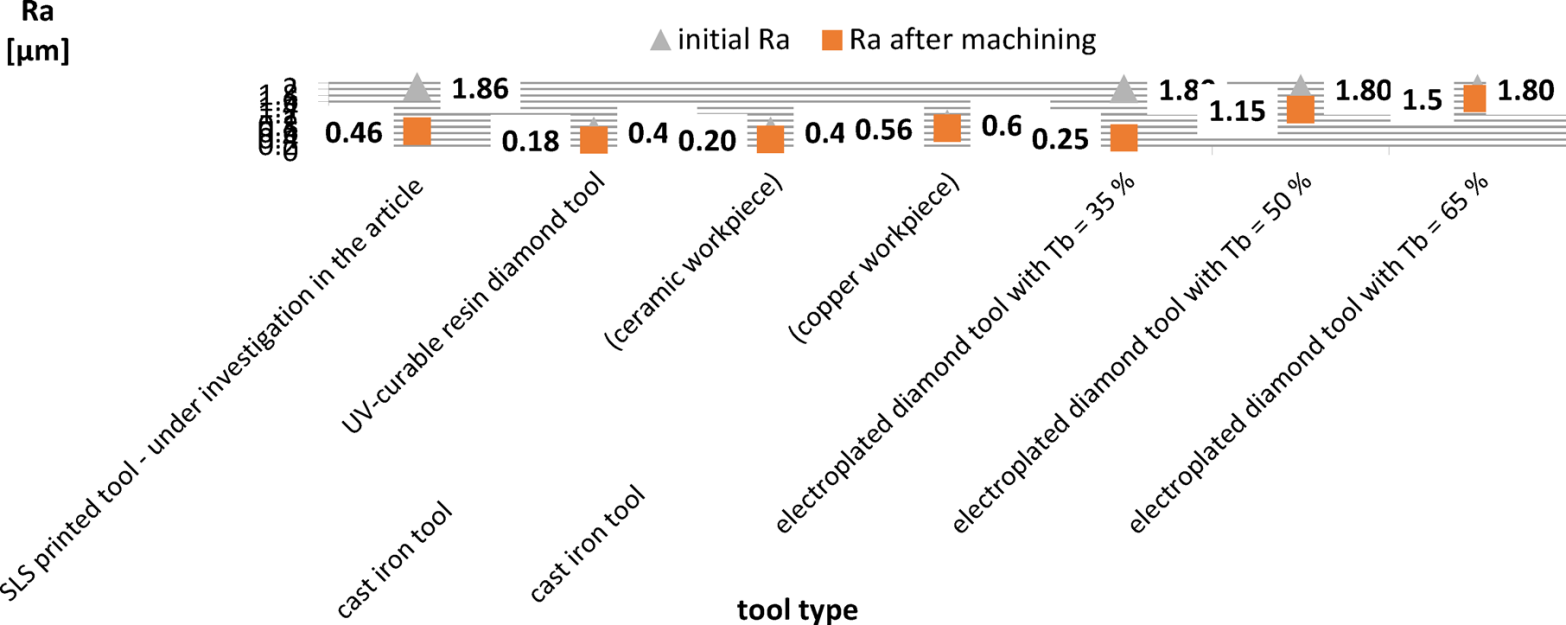
Surface roughness Ra obtained in lapping using different tools.

One of the main disadvantages of slurry-based lapping is the relatively low process efficiency, associated with the low cutting speed (*v* < 5 m/s) and excessive dosing of abrasive grains. The proposed SLS printed tool enabled to carry out an effective process of machining hard technical ceramics using a very small amount of diamond grains D107. The comparison of technological effects presented in this paper with results achieved in lapping using other metallic or non-metallic tools shows the great advantage of the proposed SLS printed tool. The process efficiency achieved for different tools and the Ra parameter are shown in Table 7; Figs. 13 and 14, respectively. The analysed abrasive machining processes and tools used, indicated in Table 7, were intended for general comparison with the lapping process using a prototype SLS-printed tool. Simultaneously, the chosen set of parameters and machining conditions for each of the compared processes and tools were adopted by the authors of the papers as those enabling the best possible technological results.

As demonstrated in Figs. 13 and 14, SLS printed tool enabled very efficient processing compared to other analysed lapping tools. According to the data given in Table 7, the weight loss of material is several times higher than in lapping with cast iron plates and UV-curable resin diamond tool^32,33^. This was due to longer processing at higher cutting speeds, unit pressure, and larger D107 diamond grains. Another advantage of the printed tool was a significant reduction in the roughness of the workpieces from Al_2_O_3_, despite the highest initial roughness indicated by Ra parameter – Fig. 14. SLS-fabricated tool and the adopted set of machining parameters enabled almost the greatest improvement in surface quality, as confirmed by the determined values of the roughness improvement rate RIR coefficient given in Table 8. The RIR coefficient considers the difference between the initial Ra and Ra after machining, divided by the value of the initial Ra roughness and expressed in %. Nevertheless, often the practical applications for ceramics elements e.g. in the optical industry, require meeting much higher surface quality requirements, which necessitates the use of additional methods of precision abrasive machining. The analysed abrasive machining process using polyamide tools can be an initial operation for precision machining ceramic materials used in industry, enabling a significant reduction in surface roughness and waviness. The obtained surface finish can be used for further fine machining processes, considering the specific quality requirements and process parameters. The undoubted advantage of the proposed solution over standard slurry-based lapping is the single dosing of the abrasive suspension only before the start of the process, which makes the machining more economical and environment-friendly abrasive process. This very limited dosage of abrasive grains with high process efficiency confirm the process with combined two- and three-body abrasion, similarly like during grinding or lap-grinding^5^. The comparison to lap-grinding with electroplated D107 diamond grains, performed also on Al_2_O_3_ ceramic samples and using the same test stand, shows that SLS printed tool enabled to obtain efficiency similar to the process carried out with electroplated tool with the nickel plating ratio *T*_*b*_ = 50%, and slightly higher than for *T*_*b*_ = 35%. As presented in Table 7, despite the high process efficiency of lapping with electroplated diamond discs, the effective machining time was only 18 min. The SLS printed lapping tool proposed in this article was used effectively by 120 min and even longer.

One of the crucial aspects of flattening precision processes is the monitoring of the tool surface, mainly its flatness condition, during the machining process. Considering the relatively low wear of the SLS-fabricated polyamide tool analysed in this article, it would be possible to continue the machining process after the 120-minute cycle or to regenerate the tool and use it in a new machining process. For this purpose, the suggested solution would be to remove the external layer of material containing fragments of abrasive grains embedded in the structure of segments with the use of cutting methods. The tool surface prepared in this way and cleaned from abrasive grains could then be given to additional conditioning operations similar to those used with conventional lapping plates. An important aspect of the proposed solution is the use of appropriate methods to assess the shape of the lapping plate, which in the case of additively fabricated tools can be successfully performed using non-contact techniques based on 3 d optical profilometers or 3 d scanners^34^. Therefore, the evaluation of the technological effects obtained with the regenerated lapping plate, including mainly the analysis of the surface quality of the tool and workpieces, may be one of the possible directions of further research.

**Table 7:** Process conditions and weight loss of material obtained in lapping using different metallic and non-metallic tools.

Tool type	Workpiece material	Abrasive Type	Process parameters		Processing time t [min]	Weight loss of material after a processing time t Δm [mg]
			Max rotational speed of a tool *n*_t_ [min^−1^]	Max unit pressure *p* [kPa]		
Segmented polyamide disc printed by SLS - under investigation in the article	Al_2_O_3_	Suspension with mixed SD 28/20 and D107 diamond grains	120	12	120	4041
UV-curable resin bond diamond lapping plate **[32]**	Al_2_O_3_	Diamond grains with an average size of 15 μm fixed in the resin bond	50	1.77	60	122
Conventional disc made of cast iron **[32]**	Al_2_O_3_	Suspension containing diamond grains with an average size of 15 μm	50	1.77	60	185
Conventional disc made of cast iron **[33]**	Tellurium copper	Paraffin and oil-based slurry with black silicon carbide grains (98 C F500)	64	3.79	90	932
Electroplated diamond disc with the nickel plating ratio *T*_*b*_ = 35% **[5]**	Al_2_O_3_	D107 diamond grains fixed in the thinnest nickel bond	60	14	18	523
Electroplated diamond disc with the nickel plating ratio *T*_*b*_ = 50% **[5]**	Al_2_O_3_	D107 diamond grains fixed in the nickel bond of the medium thickness	60	14	18	631
Electroplated diamond disc with the nickel plating ratio *T*_*b*_ = 65% **[5]**	Al_2_O_3_	D107 diamond grains fixed in the thickest nickel bond	60	14	18	741

**Table 8:** Material removal rate Dm and roughness improvement rate RIR for analysed different metallic and non-metallic tools given in Table 7.

Tool type	dm [mg/min]	RIR [%]
Segmented polyamide disc printed by SLS - under investigation in the article	33.68	75%
UV-curable resin bond diamond lapping plate^32^	2.03	60%
Conventional disc made of cast iron^32^	3.08	55%
Conventional disc made of cast iron^33^	10.36	18%
Electroplated diamond disc with the nickel plating ratio *T*_*b*_ = 35%^5^	29.06	86%
Electroplated diamond disc with the nickel plating ratio *T*_*b*_ = 50%^5^	35.06	36%
Electroplated diamond disc with the nickel plating ratio *T*_*b*_ = 65%^5^	41.17	17%

## Conclusions

The technological effects obtained during the experiments demonstrate a high potential and usability of tools produced by the SLS process. In this study the application of SLS printed tools was examined for lapping of Al_2_O_3_ ceramic samples. The most important conclusions from the conducted experimental studies, microscopic analyses and measurements are as follows:

- the SLS additive process allows developing a tool for a high-performance and environmentally friendly finish abrasive process, without any secondary post-processing;
- microscopic analysis of the worn plate surfaces confirmed the presence of diamond grains embedded into the tool’s active surface. As a result, more effective material removal along with the reduction of the surface roughness and waviness is achieved;
- lapping disc retains high cutting properties after a single dosing of the abrasive suspension only at the beginning of the process in a very small amount. The minimum quantity abrasive dosing (MQAD) proposed in the paper enables effective material removal for 120 min and even longer with;
- the SLS printed tool is able to perform an effective abrasive process of hard and brittle oxide ceramics. In general, based on microscopic observations and image analysis two-body abrasion and three-body abrasion occurred during performed experiments;
- analysis of the statistically significant results confirmed the stable course of machining;
- the developed tool was characterised by a lower wear in comparison with commonly applied resin-based and electroplated abrasive tools and at a similar level as conventional discs made of cast iron;
- the SLS method allowed for the rapid and relatively cheap (~ 200 US $) production of segmented lapping discs. Tools with smaller dimensions can be fabricated as a single and uniform element.

**Authors contribution**.

Dawid Zieliński: Writing – Original draft, Writing–Reviewing and Editing, Conceptualization, Validation, Methodology, Investigation, Data curation, Formal analysis. Mariusz Deja: Writing–Reviewing and Editing, Writing – Original draft, Conceptualization, Methodology, Investigation, Funding acquisition, Supervision. Wit Grzesik: Writing–Reviewing and Editing, Validation, Formal analysis, Visualization. **Krzysztof Żak**: Writing–Reviewing and Editing, Validation, Formal analysis, Visualization.

## Data Availability

The data that support the findings of this study are available from the corresponding author, [DZ], upon reasonable request.
